# Mathematical modeling of the socalled Allis test: a field study in orthopedic confusion

**DOI:** 10.1186/1746-1340-15-3

**Published:** 2007-01-22

**Authors:** Robert Cooperstein, Michael Haneline, Morgan Young

**Affiliations:** 1 Director of Technique and Research, Palmer West College of Chiropractic, 90 East Tasman Drive, San Jose CA 95134, USA; 2 Palmer Center for Chiropractic Research, Palmer West College of Chiropractic, 90 East Tasman Drive, San Jose CA 95134, USA; 3 Research Assistant, Palmer West College of Chiropractic, 90 East Tasman Drive, San Jose CA 95134, USA

## Abstract

**Background:**

Chiropractors use a variety of supine and prone leg checking procedures. Some, including the Allis test, purport to distinguish anatomic from functional leg length inequality. Although the reliability and to a lesser extent the validity of some leg checking procedures has been assessed, little is known on the Allis test. The present study mathematically models the test under a variety of hypothetical clinical conditions. In our search for historical and clinical information on the Allis test, nomenclatural and procedural issues became apparent.

**Methods:**

The test is performed with the subject carefully positioned in the supine position, with the head, pelvis, and feet centered on the table. After an assessment for anatomic leg length inequality, the knees are flexed to approximately 90°. The examiner then sights the short leg side knee sequentially from both the foot and side of the table, noting its relative locations: both its height from the table and Y axis position. The traditional interpretation of the Allis test is that a low knee identifies a short tibia and a cephalad knee a short femur. Assuming arbitrary lengths and a tibio/femoral ratio of 1/1.26, and a hip to foot distance that placed the knee near 90°, we trigonometrically calculated changes in the location of the right knee that would result from hypothetical reductions in tibial and femoral length. We also modeled changes in the tibio/femoral ratio that did not change overall leg length, and also a change in hip location.

**Results:**

The knee altitude diminishes with either femoral or tibial length reduction. The knee shifts cephalad when the femoral length is reduced, and caudally when the tibial length is reduced. Changes in the femur/tibia ratio also influence knee position, as does cephalad shifting of the hip.

**Conclusion:**

The original Allis (aka Galeazzi) test was developed to identify gross hip deformity in pediatric patients. The extension of this test to adults suspected of having anatomical leg length inequality is problematic, and needs refinement at the least. Our modeling questions whether this test can accurately identify aLLI, let alone distinguish a short tibia from a short femur.

## Background

Leg checking in manual medicine involves determining the relative "length" of the legs – more precisely, determining the relative position of the distal legs – in either a supine or prone patient, usually by careful observation of the location of the feet. Asymmetry in distal foot positions resulting from an actual discrepancy in the length of the lower extremities is generally called anatomical leg length inequality (aLLI). Apparent asymmetry resulting from other causes, such as unbalanced muscle function in the non-weightbearing position, is usually called functional LLI (fLLI). Many chiropractors, osteopaths, and physical therapists feel that LLI, whether structural or functional, may be a primary contributor to musculoskeletal pain and degenerative changes, both in the lower extremities and the axial skeleton. Moreover, fLLI may have diagnostic significance as well, providing evidence of subluxation or somatic dysfunction, usually in but not limited to the pelvis. Were there diagnostic significance, then reduction of fLLI would serve as an outcome measure, providing evidence of improved symmetry in body function and structure.

The clinical significance of aLLI remains controversial. With ample bodies of literature suggesting it either may or may not predict low back pain and other conditions [1], many chiropractic and other health professionals continue to exhibit interest in the matter. The oft-noted distinction of aLLI from fLLI [2,3] only adds to the complexity of the controversy. A number of excellent reviews in the chiropractic literature have not resolved the short leg question [4,5].

As investigators continue to debate the clinical significance of different types of LLI, it seems obvious that reliable and valid ways of measuring LLI are needed. There is no way to assess the clinical impact of LLI, both anatomical and functional, without having a convincing method of demonstrating it exists. Without reliable and accurate ways of measuring leg length, it would be hard to argue that leg checking should be an important part of the manual therapist's physical examination protocol. Moreover, it would be hard to conduct clinical research on whether or how LLI has adverse health consequences for patients. Since it is unlikely either the impact of or treatment for anatomic and functional LLI would be the same, the manual therapist needs a way to distinguish between the two on the shop floor. In principle, any observable LLI beyond the amount ascertained to be aLLI would by definition be categorized as fLLI.

The instrumented (i.e., objectively measured) methods that have been developed and assessed for measuring LLI include scanogram x-ray [1,6], other imaging methods (teleoroentgenogram, orthoroentgenogram, computed tomography, and ultrasound), a measurement screen [7], blocks under a standing patient to level the ilium [8,9], tape measure methods [10,11], the Chiroslide device [12], the friction-reduced table [13], and compressive leg checking [14,15]. A study conducted by Terry et al [6], comparing 3 clinical methods (two using a tape measure and one using block measurement) to scanogram x-ray, found what other investigators have found in the past: "clinical measurement of LLD [leg length discrepancy] may be grossly inaccurate."

Although it would certainly be worthwhile to continue developing and assessing instrumented methods of identifying LLI, it is also necessary to develop and refine non-instrumented (visual and manual) methods. Although these less technologically developed methods *may *eventually be found less accurate (that will not be known until the work is done), they have the advantage of easier implementation and thus are more clinically relevant. As both Knutson [2] and Cooperstein [16] point out, the exact magnitude of a short leg may be less important than knowing its side, and perhaps a quick judgement as to whether the magnitude appears large or little. These less ambitious measurement methods may be all that is required from a clinical point of view.

In the chiropractic profession, the primary leg checking procedures that are done include a variety of both supine and prone procedures, including the rather elaborate Derifield leg check [17-19]. Another method that is occasionally used is commonly described as the "Allis test." The purpose of the present study is to perform mathematical modeling of this so-called Allis test, test under a variety of hypothetical clinical conditions, prior to undertaking a clinical investigation.

Despite the fact that the reliability of prone and supine leg checking procedures is reasonably well-known [2], and there have been some studies on their validity [14,15], we are not aware of any investigations of the Allis protocol for determining aLLI.

In our search for information on the Allis test, nomenclatural and procedural issues became apparent, as explained in the Discussion section below. The Allis test as we performed it is discussed in the Methods section.

## Methods

**Figure 1:**
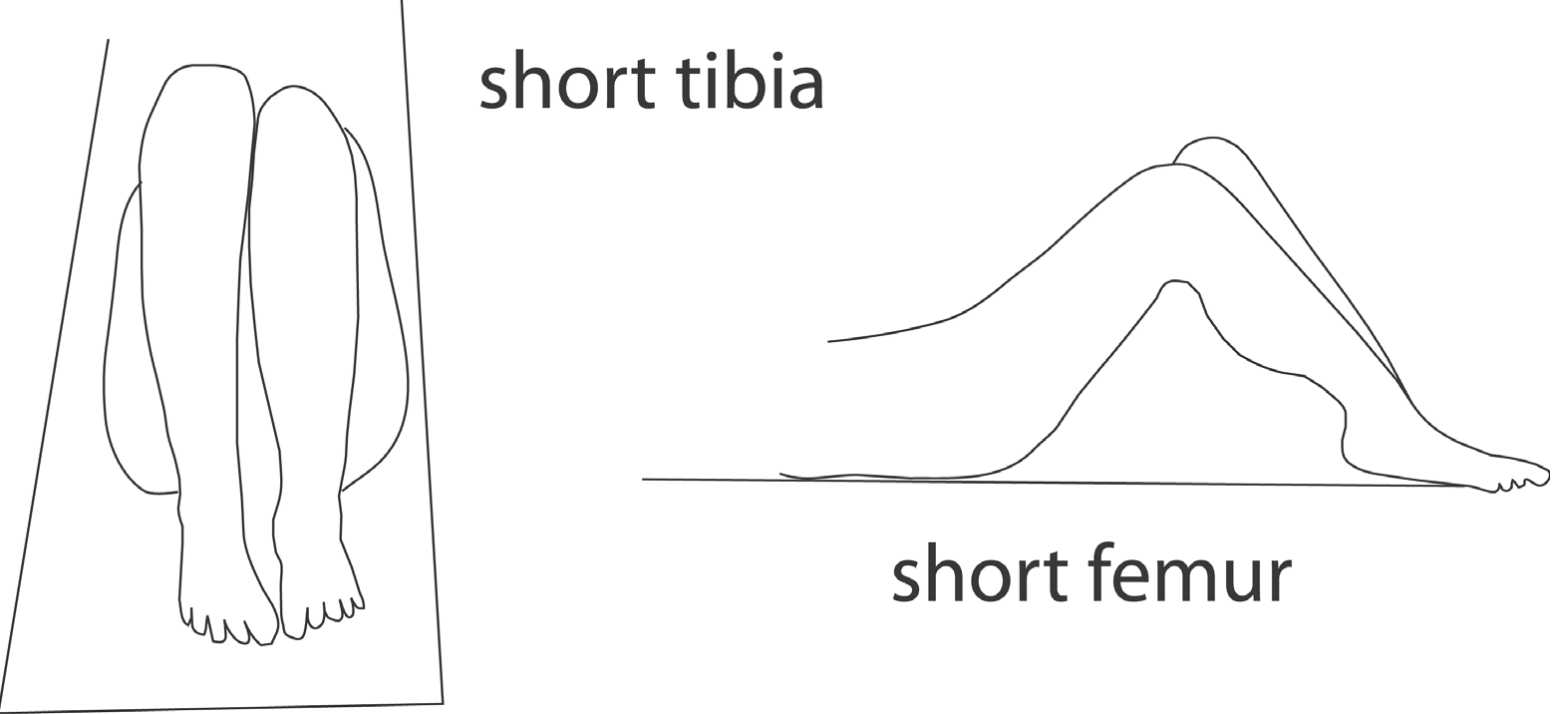
**The so-called Allis test**. So-called Allis test, as it appears in typical textbooks used in chiropractic colleges.

To perform the Allis test for aLLI, a subject is carefully positioned in the supine position, with the head, pelvis, and feet centered on the table. After an assessment for anatomic leg length inequality, the knees are flexed to approximately 90°. The examiner then sights the short leg side knee sequentially from both the foot and side of the table, noting its relative locations: both its height from the table and its Y axis position. Observed from the foot of the table, a low knee on the short leg side is said to identify an anatomically short tibia. Observed from the side of the table, a more cephalad knee is said to identify an anatomically short femur. (See figure 1.)

**Figure 2:**
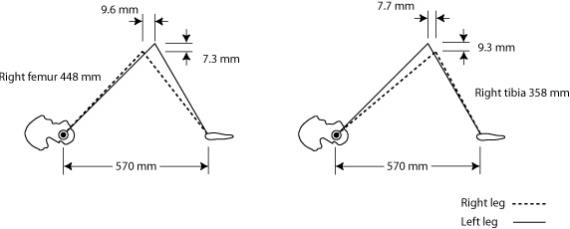
**Schematic changes in knee location for 12 mm length reductions**. Schematic changes in knee height and Y axis location for 12 mm shortening of femur (left) or 12 mm shortening of tibia (right). Not drawn to scale.

**Figure 3:**
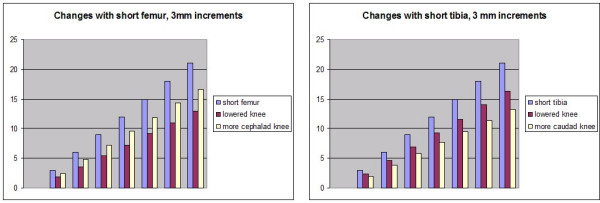
**Changes in knee location for 3 mm incremental length reductions**. Predicted changes in knee height and Y axis location for 3 mm incremental changes in femur or tibia.

For the purpose of mathematical modeling the Allis test, we assumed a left tibial length of 370 mm, a left femoral length of 460 mm, and a distance from hip to foot of the supine patient of 570 mm. These numbers are based on a typical tibio/femoral ratio of 1 to 1.26 [20], and a knee angle of approximately 90°. Then, the femur, tibia, and hip-foot distances created a triangle of known dimensions (see figure 2), allowing the use of trigonometry, such as the law of cosines, to calculate the 3 angles of the triangle. Knowing these angles, we further calculated changes in the location of the right knee that would result from hypothetical reductions in the length of either the tibia or the femur. We arbitrarily chose 12 mm for the amount the tibia and/or femur were changed (figure 2) although we also portray the consequences of incremental 3 mm changes (figure 3). These changes in knee position affected both the altitude of the knee from the table top, and its Y axis position along the length of the table. We also calculated the hypothetical effect of one hip being drawn up cephalad compared with other, while the feet were kept even at the foot of the table, thus increasing the hip-foot distance on one side. Finally, we calculated the effect on knee location when the femur was shortened and the tibia lengthened by the same amount (in essence, a change in the tibio/femoral ratio), thus leaving the overall length of the leg unchanged, and vice versa.

## Results

Our results are shown in figures 2 and 3 as well as in table 1, which assumes 12 mm changes in each experimental condition. The knee altitude is diminished with *either *femoral or tibial length reduction. The knee is shifted cephalad when the femur is reduced in length, and caudally when the tibia is reduced. Shortening of the femur has an approximately 25% greater impact on knee Y axis location than tibial shortening, in the opposite direction; tibial length reduction results in an approximately 25% greater drop in knee altitude than femoral shortening.

**Table 1:** Summary of experimental conditions and changes in knee location. In mms. Positive values represent knee movement in the cephalad direction and increased height. Negative values represent knee movement in the caudad direction and decreased height.

	**experimental condition**	**tibia**	**femur**	**hip-foot distance**	**Δ knee ht.**	**↑ knee cephalad**
**left leg**	no change	370.0	460.0	570.0	n/a	n/a
**right leg**	short femur	370.0	448.0	570.0	-7.3	9.6
**right leg**	short tibia	358.0	460.0	570.0	-9.3	-7.7
**right leg**	tibia ↓ femur ↑	358.0	472.0	570.0	-2.3	-17.5
**right leg**	femur ↓ tibia ↑	382.0	448.0	570.0	1.8	17.5
**right leg**	hip cephalad 12	370.0	460.0	582.0	-5.6	7.4

Although we do not represent it graphically, we calculated the impact on knee location of changes in the femur/tibia ratio, while the length of the lower limb was not changed overall. Depending on which bone was shortened, the knee height either increased or decreased slightly; however, the knee's Y axis location was hugely impacted, made more caudad by tibial length reduction and more cephalad by femoral length reduction. As shown in table 1, increasing the tibia by 12 mm and decreasing the femur by 12 mm moved the knee 17.5 mm cephalad, and reversing the changes moved the knee 17.5 mm caudad. Finally, we also calculated the consequence of one hip being drawn cephalad on the table (while the foot remained in the same position), due to careless patient positioning, asymmetry of lumbopelvic muscle tone, soft tissue contractures, hip misalignment, or any other mechanism. This lowered the knee by 5.6 mm and brought it more cephalad; e.g., a 12 mm hip retraction lowered the knee by 4.6 mm, and brought the knee 7.4 mm more cephalad.

## Discussion

Before discussing our quantitative results and their implications, we must first address certain nomenclatural issues that came up during the execution of this project. Versions of the Allis test, as seen in figure 1, can be found in several textbooks commonly used by chiropractic students: Hoppenfeld p. 165 [21], Haldeman p.294 [22], and Magee p.246 [23] as well as included within innumerable course notes among the chiropractic colleges. The test has been mislabeled and misapplied by a number of authors, producing confusion in the literature which apparently has not been unrecognized. Accordingly, we attempt to document some of these inaccuracies in the following section.

The original test named after the historical Dr. JB Allis (c. 1960) is quite different from the test we modeled, and was apparently restricted to children or even infants, purporting to identify gross deformity such as congenital hip dislocation, hip dysplasia, tibial bowing, or marked anatomical leg length inequality.

**Figure 4:**
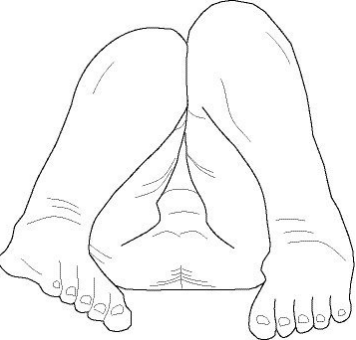
**Allis/Galeazzi test or Sign, in orthopedic medicine**. The Allis/Galeazzi test or sign identifies gross hip or other lower extremity deformity in children, usually infants.

According to Stricker and Hunt [1], the Allis test can discriminate between a short femur or tibia in children, if there is a suspicion of aLLI. They write: "If a LLD [leg length discrepancy] is suspected by pelvic tilt during standing, the location of discrepancy may be verified by performing the Allis test and the reverse Allis test. The Allis test (also called Galeazzi test) is performed in the supine patient by noting relative knee heights when both hips and knees are flexed 90°. This will determine how much discrepancy is located in the thigh segment. The patient is then turned prone with the knees and ankles at 90° (and both hips in neutral rotation) to determine how much LLD is present below the knees." Based on our modeling, as well as simple inspection of figure 4, it is not entirely clear why Stricker et al believe that knee height discrepancy assessed from the foot of the table confirms femoral deficiency, as compared with tibial deficiency. In another paper [24], Stricker's depiction of the Allis/Galeazzi test does not conform to his own stipulation that the hip and knee are flexed to 90°; our figure 4 is based on this depiction. A similar illustration appears in an article by Leet and Skaggs [25], who state: "The test is positive when the knees are at different heights as the patient lies supine with ankles to buttocks and hips and knees flexed." Their illustration does not really appear to bring the infant's feet to his or her buttocks.

**Figure 5:**
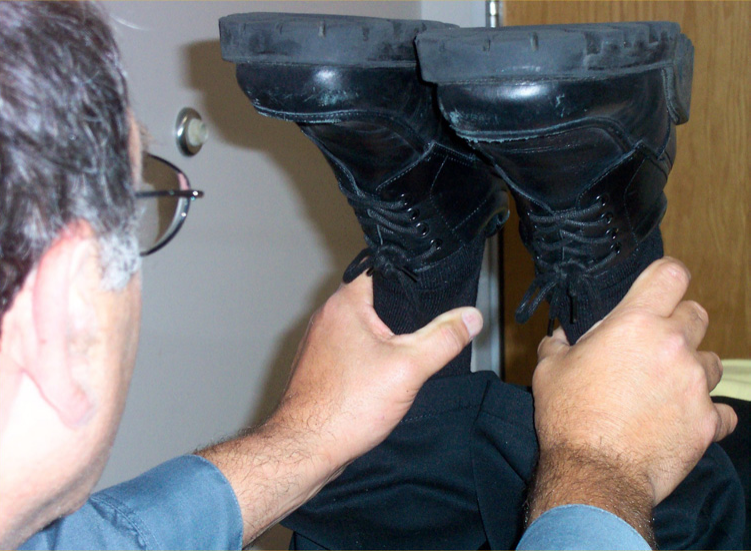
**Assessing tibial length asymmetry, using malleoli as landmarks**. The relative length of the tibias can be assessed by carefully comparing the elevation of the medial malleoli.

**Figure 6:**
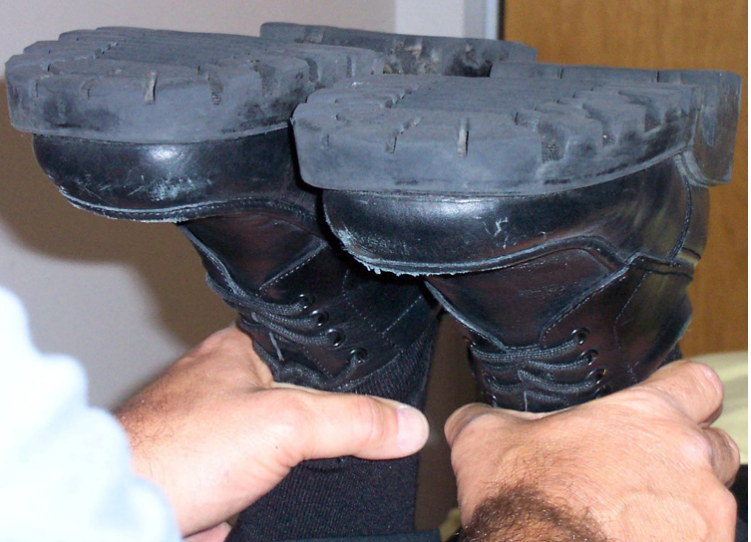
**Assessing tibial length asymmetry, using counters of shoes as landmarks**. The relative length of the tibias can be assessed by carefully comparing the elevation of the shoe counters.

In neither of these papers do we see any mention of assessing the Y axis location of the knee, as chiropractors performing their version of Allis are wont to do, and as is described in several textbooks commonly used by chiropractors. It might be added that Stricker's ancillary prone, knees-flexed procedure for identifying tibial length discrepancy can also be found in Peterson et al [26], p.322. This is portrayed in figures 5 and 6. Cooperstein has devised a model in which tibial length discrepancy in this position may be apparent (i.e., functional) rather than structural, the result of a difference in the stiffness of the anterior thigh musculature [17,18].

In the 1987 first edition of Magee's orthopedics textbook [23], the index lists page 225 for the Allis test, but there is nothing on or near that page pertinent to anything like it. Page 255 of the same text depicts "Galeazzi's sign (Allis test)," with an illustration like the left side alone of our figure 1, stating it is "good for assessing unilateral dislocation of the hip only and may be used in children from 3 to 18 months of age. Page 246 of the same text provides an illustration nearly identical to our figure 1 (both left and right sides), purporting to identify "leg length discrepancy." We are not able to easily reconcile the information provided on pages 255 and 246 of this 1987 text. The 2002 4^th ^edition of Magee's text [27] contains the same inconsistency, on pages 627 and 628.

Magee also describes another procedure he calls the "Weber-Barstow maneuver" ([27], p.629) for assessing LLI, that superficially resembles Allis/Galeazzi. We found other internet references to the Weber-Barstow procedure, such as course notes from the University of Minnesota [28] and another from the University of Maryland [29]. *(Although these course notes were available when accessed on July 20, 2006 and August 25, 2005 respectively, their URLs had become inoperable by the time the present article was in press.)*  Further researching showed the proper name for this Allis-like test is the "Wilson-Barstow maneuver," as described by Donatelli ([30] p.412). Dorman portrays and discusses a procedure he also calls the Wilson-Barstow procedure [31], but he adds motion testing and thus winds up showing something quite different from Donatelli.

It is hard to escape the impression that the literature on the Allis/Galeazzi/Wilson-Barstow tests is very confusing and inconsistent. We do not know why, how, or when a simple visual test developed to assess gross structural deformity (such as congenital hip dislocation or dysplasia) mutated into a test for LLI in adults, possibly of small magnitudes. It appears that writers of orthopedic textbooks and their invited authors are making liberal use of each other's writings, without critically evaluating the accuracy of their attributions or validity of the tests. It is common to find discrepancies between the words authors use to describe test procedures, and the illustrations that appear in their texts.

Irrespective of nomenclature, our modeling shows that the test shown in figure 1 (the so-called Allis test in chiropractic, and apparently unnamed in orthopedic medicine) is flawed. Although it may detect aLLI, this test as commonly construed, as a differential diagnosis of short femur vs. short tibia, is not likely valid. It is simply not the case that a low knee seen from the foot of the table suggests a short tibia, whereas a cephalad knee seen from the side suggests a short femur. On the contrary, either a short tibia or a short femur would likely lower the knee as seen from the foot of the table. In addition, a cephalad knee likely suggests a short femur, and a caudad knee a short tibia, when the short leg is sighted from the side of the table. However, the accuracy of such a determination would in turn depend on a series of other factors that would affect the knee position as seen from both from the side and foot of the table:

• The hips would have to be in the same Y axis position. Table 1 shows that cephalad displacement of one hip results in a somewhat lesser degree of cephalad knee displacement. It is not obvious how an examiner would confirm symmetric hip placement on the table.

• The tone and/or stiffness of the gluteal muscles would have to be the same or similar, since this could affect the relative position of the femoral heads. Cooperstein's model of the Derifield pelvic leg check [17,18] invoked similar differences in the stiffness of the anterior thigh musculature to explain differences in apparent tibial length.

• Equal and opposite differences in tibia and femur lengths would create asymmetry in both the Y axis hip locations and in knee height, as seen in table 1. Thus, the so-called Allis test would suggest anatomic LLI where it is not present, generating a false positive result

## Conclusion

The Allis/Galleazzi test described in pediatric orthopedic medicine is not performed identically to the so-called Allis test as commonly used in chiropractic, a test which can also be found in some orthopedic textbooks as an unnamed procedure (figure 1). Our modeling questions whether this test can identify aLLI, distinguish between a short tibia or short femur, or avoid false positives in cases where there are equal but opposite discrepancies in the length of the femur and tibia. Depending on the use to which the information is to be put, it may not be very important to distinguish a short femur from a short tibia; the manual therapist is presumably more interested in total limb length, than the differential diagnosis suggested by the test we studied.

This study is limited by the fact that it is pure modeling, and a clinical study will be needed to see if its predictions are borne out. The simple stick figures we used in figure 2 are not necessarily an entirely appropriate representation of a flesh and blood leg, given the complexity of its joint kinematics. Future studies may address the interexaminer and intraexaminer of this type of visual check, and compare its results against an accepted gold standard for aLLI, such as the scanogram x-ray.

It would be naive to assume orthopedic specialists and other authors always carefully read and consider every test in each other's textbooks and articles; sometimes what seems scholarly is merely convention that has mutated over many editions and authors. This may be due to an attempt at being more "complete" rather than striving to be correct. Even authoritative references are not above reproach. That is, mistakes occur, and often propagate through the literature. Our point is not to demean other authors but rather promote critical thinking in appropriating information.

## Abbreviations

LLI: Leg length inequality

aLLI: anatomical leg length inequality

fLLI: functional leg length inequality

LLD: leg length discrepancy

## Competing interests

The author(s) declare that they have no competing interests.

## Authors' contributions

RC conceived of the study, performed the trigonometric calculations, and wrote the first draft of this publication. MH helped produce the illustrations and author the manuscript. MY did most of the literature searching related to the Allis/Galeazzi test, and article retrieval, and helped author the manuscript.
